# A Randomized Controlled Trial on Mutual Support Group Intervention for Families of People With Recent-Onset Psychosis: A Four-Year Follow-Up

**DOI:** 10.3389/fpsyt.2018.00710

**Published:** 2018-12-18

**Authors:** Wai Tong Chien, Daniel Bressington, Sally W. C. Chan

**Affiliations:** ^1^The Nethersole School of Nursing, Faculty of Medicine, The Chinese University of Hong Kong, Shatin, Hong Kong; ^2^School of Nursing, Faculty of Health and Social Sciences, The Hong Kong Polytechnic University, Kowloon, Hong Kong; ^3^School of Nursing and Midwifery, University of Newcastle, Callaghan, NSW, Australia

**Keywords:** mutual support group, family intervention, recent-onset psychosis, psycho-education, randomized controlled trial

## Abstract

**Introduction:** Recent research in Western countries has indicated that family interventions in schizophrenia and other psychotic disorders can reduce patient relapse and improve medication compliance. Few studies have addressed Chinese and Asian populations. This study tested the long-term effects of a 9-month family-led mutual support group for Chinese people with schizophrenia in Hong Kong, compared with psycho-education and standard psychiatric care.

**Methods:** A randomized controlled trial of Chinese families of patients with recent-onset psychosis (≤5 years of illness) was conducted between August 2012 and January 2017, with a 4-year follow-up. Two hundred and one Chinese families of adult outpatients with recent-onset psychosis were randomly selected from the computerized patient lists and randomly assigned to either mutual support, psycho-education, or standard care group (*n* = 70 per group). Family caregivers were mainly the parent, spouse, or child of the patients. Mutual support and psycho-education group consisted of 16 two-hour group sessions and patients participated in three sessions. The standard care group and the two treatment groups received the routine psychiatric outpatient care.

**Results:** Patients and families in the mutual support group reported consistently greater improvements in overall functioning [family functioning, *F*_(2, 203)_ = 8.13, *p* = 0.003; patient functioning, *F*_(2, 203)_ = 6.01, *p* = 0.008] and reductions in duration of hospitalizations [*F*_(2, 203)_ = 6.51, *p* = 0.005] over the 4-year follow-up. There were not any significant increases of medication dosages or service use by both the family support and psycho-education groups over time.

**Conclusions:** The peer-led family support group can be an effective psychosocial intervention in early psychosis indicating long-term benefits on both patient and family functioning and re-hospitalizations.

**Clinical Trial Registration:** NCT00940394: https://register.clinicaltrials.gov.

## Introduction

Psychosis is a severe mental disorder often first occurring in adolescents and young adults. Disturbing psychotic symptoms such as hallucinations, delusions and disorganized thoughts, contribute to some loss of contact with reality. A psychotic illness can cause high levels of psychological distress in patients themselves and often results in a substantial burden of care for their families and health care services (1, 2). More than 50% of people with psychosis, especially those in the early stage of the illness, are living with and being supported by their family members during their community-based treatment and rehabilitation (3–5).

Family caregivers often neglect their physical and psychosocial needs because they spend a great deal of time being concerned about their ill relative's mental health and psychiatric treatment. Family uncertainties about the course and prognosis of their relative's illness and potential for recovery can also trigger high levels of anxiety, tension and stress, contributing to caregiver burden (5). Family caregivers need to adjust and adapt to the new role of informal carer, but they often feel unprepared or unequipped to appropriately care for their mentally ill relative. Family intervention is based on the assumption that a stressful interpersonal environment within the family context can exacerbate psychotic symptoms and cause premature or frequent relapses. Therefore, this approach to intervention is highly recommended as one of the core treatment strategies in illness management and is included in the best practice guidelines for psychosis (6, 7).

Recent systematic reviews of controlled trials suggest that evidence-based family interventions in psychotic disorders consist of different combinations of psychotherapeutic strategies, mainly including psychoeducation, stress management, cognitive appraisal, and problem solving skills. These reviews show that such family-based interventions can improve psychotic patients' mental condition and medication/treatment adherence, and significantly reduce their risks of relapse and re-hospitalization over a medium long-term (e.g., 12–18 months) follow-up (8–10). A few randomized controlled trials in Western countries (e.g., in the U.S. and U.K.) indicated significant positive results of psychoeducation and other family interventions for psychosis in reducing relapse rates and improving social functioning over 1–2 years post-intervention (8, 9). Practice guidelines for early psychosis (6, 10, 11) and related systematic reviews (8, 12) also recommend that the families should be provided with family-oriented psychosocial interventions to support their caregiving endeavors and improve functioning, mental health, and well-being for themselves. However, the effects of family interventions on caregivers' outcomes are less established in comparison to those of the patients. For example, most family intervention research focuses on outcomes concerning patients' enhanced knowledge of schizophrenia and its treatment, reduced relapse rates and re-hospitalizations, and improved medication adherence (9, 12, 13).

Only a few studies have reported that family interventions improve caring experience, caregiving burden and family functioning in recent-onset or early-stage psychosis (e.g., ≤5 years of illness). Several of these controlled trials have also shown positive results of psychoeducational programs for families of people with psychosis in families' social functioning and caregiving burden up to 12 months follow-up (14–16). Whereas, inconsistent and modest effects are seen in other family psychosocial health outcomes. While most family interventions have measured a variety of study outcomes, these controlled trials and their intervention programs/protocols often focus on patients' rather than caregivers' health outcomes (8). A few recent controlled trials of different approaches to family intervention for people with psychotic disorders reported that family caregivers' psychological distress and patients' mental state were significantly improved. However, other important family and patient outcomes, such as self-care and psychosocial functioning, perceived social support and health-related quality of life, or their long-term effects (i.e., >2 years), were inconclusive or resulted in only small-sized effects (14–17).

Peer-led mutual support groups that emerged in the 1990s are less structured supportive educational group intervention programs facilitated by peers with similar life situations to the group members, such as people with chronic physical and mental health problems, and their family caregivers (18, 19). Over the past two decades, there are increasing studies of peer-led or peer-facilitated mutual support groups for family caregivers who had lived experiences of caring for a family member with a psychotic disorder and other severe mental illnesses (18). Peer support for patients with psychosis and their family members is now considered to be an integral part of the self-help and/or empowered social movement on the care of people with severe mental illnesses. Whereas, peer support groups specifically for family caregivers of people with psychosis are designed to provide caregiving support and meet the health needs of caregivers that are not addressed by routine mental health care services (19). Previous studies have shown that clinician- and peer-facilitated mutual support groups for family caregivers of people with severe mental illnesses can reduce caregiving burden and improve the family's knowledge and stress management. However, they appear less effective in improving patient's symptoms and both the patient's and family's functioning and adaptive coping (19–21).

Family- or peer-led mutual support groups require less intensive training of family peer leaders in psychoeducation, coping and stress management skills. These peer-led support groups offer a flexible, interactive client-directed approach for family caregivers to cope with their caregiving burden and provide an opportunity for model learning of caregiving skills from peer caregivers (21, 22). In addition, these family support groups focus on improving caregiving attitudes, emotions and empathy, as well as promoting effective communication and relationships between family caregivers and their patients (19, 21). Despite some research evidence supporting the positive effects of family-led support groups in severe mental illness, these studies are mainly non-randomized controlled trials and contain inadequate methodological rigor to confirm their clinical efficacy for families of people with psychosis. The methodological limitations of the earlier studies include small sample sizes, high drop-out and/or program in-completion rates (i.e., 20–60%), inadequate training of caregiver interveners, limited intervention fidelity, and mainly short to moderate term (i.e., 3–12 months) follow-ups (19, 22, 23). In addition, there are only limited numbers of family intervention studies that focus on Chinese and Asian populations (19, 24).

We conducted an earlier randomized controlled trial (25) of a 14-session family-led mutual support group for 45 families caring for Chinese patients with early-stage schizophrenia (<5 years of illness) in two outpatient clinics in Hong Kong. The results demonstrated that the family-led support group program could be a more effective community-based psychosocial intervention for Chinese psychotic patients than routine psychiatric care in improving patients' mental state and duration of psychiatric re-hospitalizations, as well as their families' functioning and perceived social support over 12- and 24-month follow-up. The participants in the family-led support groups also showed significantly greater improvements in family and patient functioning at the 24-month follow-up than those in the professional-led psychoeducation groups. However, our earlier study (25) and a few similar trials (19, 20, 22, 24) had recruited small non-representative samples of people with long-term psychotic illnesses and utilized health professionals as co-leaders or facilitators. Consequently, it is uncertain whether the full family-led intervention (with structured training and preparation for the caregivers as leaders and clinicians as supporting resource persons) can be applied successfully to early-stage psychotic patients with a Chinese family-oriented culture. In assisting Chinese families whose family member suffers from a psychotic disorder, it is important to acknowledge and make use of the culture-specific family structure, roles, and process in a group program. These Chinese cultural tenets include having a strong sense of interdependence with collective identities and family-based decision making. Chinese people also have high expectations of family members to study, work and live in a “proper” manner, and exhibit a unique sense of filial responsibility, respect and harmony between generations (19, 26). Therefore, this randomized controlled trial aimed to evaluate the effectiveness of a peer-led family mutual support group intervention for Chinese patients with recent-onset psychosis (i.e., <5 years of the illness) on a variety of family caregiver and patient health outcomes over a 4-year follow-up, when compared with a conventional family psychoeducation group program and those with treatment-as-usual only.

## Methods

### Study Site and Participants

This randomized controlled trial adopted a single-blind, parallel groups randomized controlled trial with repeated-measures, three-arm design. The controlled trial was conducted at three regional psychiatric outpatient clinics in Hong Kong (i.e., one regional outpatient clinic in each of three main geographical regions—New Territories, Kowloon, and Hong Kong Island) between August 2012 and January 2017. It is part of our multi-center randomized controlled trial registered in ClinicalTrials.gov (Ref.: NCT00940394), but the sample in this study were people primarily diagnosed with recent-onset psychosis (whereas, the samples in the registered clinical trials included all types of schizophrenia spectrum and psychotic disorders with varied duration of illness). There were around eight clinics in each of these three regions serving about 60,000 adult outpatients with psychotic disorders in Hong Kong. Eligible pairs of psychotic patients and family caregivers were randomly assigned to one of three treatment conditions: family-led mutual support group (FMSG), family psycho-education group, or treatment-as-usual only (TAU). There were no deviations from, or violations and amendments to the trial protocol after commencement.

All participants were assessed by an independent assessor (a trained research assistant who was blinded to treatment allocation) at recruitment/baseline (Time 1) and one-week (Time 2), 12-month (Time 3), 24-month (Time 4), and 48-month (Time 5) after completing the interventions. During the 4-year follow-up, all participants did not receive any other family support group intervention unless it was held and run by the FMSG members themselves. The main hypotheses were that a family-led mutual support group (FMSG) participants could significantly improve caregivers' family functioning and patients' re-hospitalization rates (i.e., primary outcomes), reduce family burden and patients' psychotic symptoms and functioning, as well as improve their utilization of mental health services, when compared with those in family psychoeducation group or TAU only.

**Figure 1:**
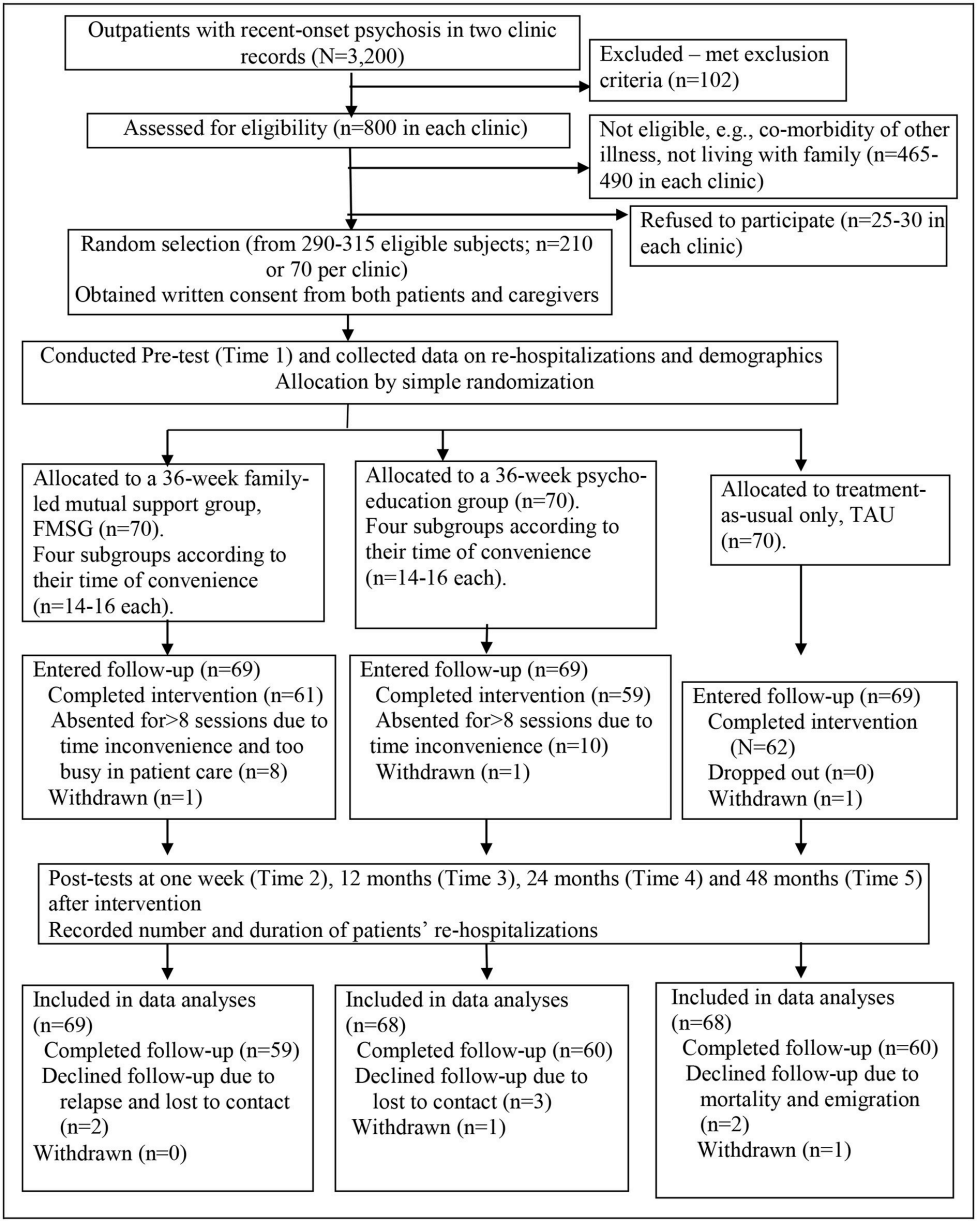
Flow diagram of the controlled trial procedure. The figure shows the main procedure of this randomized controlled trial according to the latest CONSORT statement (27). Families of patients with recent-onset psychosis in three outpatient clinics (*N* = 800 per clinic) were assessed and 290–315 of them were found eligible in this study. Two hundred ten of them (70 per clinic) were randomly selected and after baseline measurement, were then randomly allocated into one of the three study groups [Family-led Mutual Support Group (FMSG), Family Psychoeducation Group, or Treatment-As-Usual (TAU) only; 70 subjects per group]. After completion of the interventions, majority of them in the FMSG, Family Psychoeducation Group and TAU (*n* = 69 in each group) continued to be followed up for 48 months and only a few lost/withdrew from the study. On intention-to-treat basis, 69 in the FMSG and 68 in the other two study groups were included into final outcome analysis.

Participants were randomly selected from the patient lists of the three clinics, consisting of around 3,200 patients diagnosed with recent-onset psychosis (18% of the total patient population), using computer-generated random numbers. Potential subjects with an appropriate psychiatric diagnosis were assessed by the psychiatrists to ensure that they were mentally fit to take part in the study and were then referred to the research assistant as appropriate. The research assistant then assessed the referred potential subjects based on the inclusion and exclusion of this trial. Figure 1 summarizes the subject enrolment and flow of the trial procedure according to the latest version of the CONSORT statement (27).

To be eligible, patients who were Hong Kong Chinese residents and one main family caregiver had to meet the following inclusion criteria: (a) the caregivers were living with and taking care of one family member (the patient) primarily diagnosed with recent-onset psychosis (<5 years) at recruitment, according to the criteria in the 4th revised edition of Diagnostic and Statistical Manual for Mental Disorders, DSM-IV-R (5); and (b) both were aged 18 years or above and could understand Mandarin or Cantonese. Exclusion criteria included those caregivers who had history of mental illness themselves (*n* = 22), or who had been the primary caregivers for <3 months (*N* = 30); and those patients who were illiterate, with co-morbid cognitive disorders, learning disability or personality disorders, and/or participated (were participating or scheduled to participate) in any other psychosocial intervention program(s) within the last 6 months.

Of 800 (57%) potential subjects in each clinic accessible for screening by the research assistant, 290–315 (36–39%) who were found eligible in the three clinics agreed to participate; and 25, 28 and 30 (3–4%) refused to participate. Major reasons for their refusals included lack of time and/or interest to participate, concerns about being stigmatized or discriminated if they participated, and difficulty in asking others to help in taking care of the patient when participating in the intervention. From all eligible subjects, 70 were randomly selected independently from a patient list of each clinic (22–24%), using the randomization procedure recommended by the National Health/Medical Research Council Clinical Trials Center (28). In each of the three clinics, all eligible subjects were listed in alphabetical orders of patient surnames. Three sets of computerized random numbers were generated by a statistician who was blinded to the recruitment procedure to match with the three patient lists per clinic.

Ethical approval to conduct this trial was granted by the NTEC-CUHK Clinical Research Ethics Committee, and informed written consent was obtained from all individual participants by a research assistant before completing baseline measurement and randomization. Their identity and data confidentiality and right to refuse/withdraw from study participation were assured. For those patients with more than one carer, the family caregiver who had the majority of the caring role (as indicated by the patient) was recruited. The families were then randomly assigned to one of the three arms: FMSG, family psychoeducation group, or TAU only (*n* = 70 per group).

### Sample Size Calculation

Sample size estimation was performed in relation to the main hypotheses and (primary) outcome variables of this controlled trial. It was calculated that 62 families per trial arm would be needed to detect a difference on change in mean score of family functioning for 1.6 points, with a standard deviation of this score change of 3.5 (study power of 0.8, *p* < 0.05). This can result in a moderate effect size of 0.44 according to the findings of two systematic reviews of family mutual support groups for people with psychotic disorders (19, 22). Referring to two randomized controlled trials of family mutual support group and psychoeducation programs for patients with schizophrenia (24, 25), the effect sizes of patients' re-hospitalization rates and family functioning ranged between 0.42 amd 0.68 at 6-month and 12-month post-intervention, thus requiring for 42 to 68 subjects to detect a significance difference between groups. Taking account of about 15% attrition rate found in our previous controlled trials of mutual support and psycho-education group interventions up to 24 months follow-up (25, 29), 210 subjects/families (70 per arm) could detect statistical differences on family functioning and patient re-hospitalization rates between three arms at moderate effect size of 0.42 (i.e., the lowest value from the above studies) and 80% power (two-tailed, *p* < 0.05).

### Random Assignment of Study/Intervention Groups

After baseline measurement in their attending clinics, the 210 randomly selected caregiver-and-patient dyads were randomly allocated to one of three study groups (*n* = 70 per arm) with three sets of computerized random numbers generated by an independent statistician using a stochastic minimization program (30) to balance caregivers' gender and patients' symptom severity between groups. To reduce subjective bias and/or treatment contamination, the participant list was locked away and concealed from the researchers, outcome assessor, and clinic staff. The researchers also requested and reminded all participants not to discuss their group allocation with health care staff, or co-patients and family caregivers in the clinics. One research assistant and one researcher (first author) who were blinded to group assignment performed the outcome assessments (and data entry) and checking of data entry accuracy, respectively.

### Treatments

The family-led mutual support group (FMSG) program (in addition to routine psychiatric outpatient care) consisted of 16 bi-weekly 2 h sessions co-led by two peer family caregivers. These caregivers were relatively more experienced in caregiving and were trained by the researchers to perform the peer leader role with a three full-day psychoeducation and supportive skills workshop. The peer leaders worked closely with two resource persons (the first author and one rehabilitation nurse specialist) who offered assistance for group resources, group development in stages and services referrals. The workshop's contents were based on similar program protocols and the researchers' intervention programs of family mutual support groups in psychotic disorders (20, 24, 25, 31). Table 1 outlines the contents of 16 group sessions in terms of five stages. The sessions placed emphasis on supportive sharing and information exchanges, problem-solving and caregiving skill practices during and after sessions. Important Chinese family culture issues were also covered and discussed (e.g., stigma in relation to mental illness and help-seeking, interdependent family process and collective decisions and actions, harmonious and respectful family relationships, and preference to practical and instrumental help and assistance) (19, 26, 32, 33).

**Table 1:** Outline of main content of family-led mutual support group program^a^.

**Stage**	**Goals**	**Content**	**Length of each stage ^***b***^**
Engagement (Introduction and orientation)	• Establishing mutual trust and respect • Setting up common goals and action tasks	• Orientation to family group intervention and establishing trust and acceptance among members • Introducing and negotiating leader and member roles and responsibilities and ground rules • Negotiation of goals, objectives and action plans of individual sessions • Initial discussion of psychosis and its impacts and learning on family	2 sessions
Awareness and addressing mutually shared psychosocial needs	• Openly sharing, understanding and showing respect and support on individual concerns and demands for caregiving • Exploration of cultural issues in families	• Power resolution for control or dominance, mistrust, decision-making among group members • Openly sharing of challenges, intense emotions and distress in caregiving, and family interactions, and learning about mutual support and acceptance • Information sharing of psychosis and its related problem behaviors and impacts • Discussing important issues in Chinese family culture and their relations with psychosis (stigma concerning mental illness and related help-seeking, interdependent family process, collective decisions and actions, harmonious and respectful family relationships) • Discussing the ways to manage negative emotions and ignorance to their patient	4 sessions (patient included in one session)
Managing common and individual physical and psychosocial needs of self and family members	• Understanding and addressing about most important needs for themselves, patient and the whole family	• Discussion about each member's physical and psychosocial health needs • Information of medication (and its adherence), stress and illness management strategies, and available and accessible mental health services • Effective communication skills with patient and other family members and seeking social support from family members and others • Establishing home management strategies e.g. finance and budget, environment and hygiene	4 sessions (patient included in 2 sessions)
Taking up caregiving roles and demands and facing with challenges	• Learning from peer members and group leader about coping and problem solving skills in caregiving and managing life situations	• Sharing of coping skills and mutual support for skill rehearsals/practices • Enhancing problem-solving by working on individual patient and family life situations or problems • Behavioral rehearsals of interpersonal interactions, especially with patients, and giving comments and support on practices within group • Practicing their learned coping skills to real family situations (in-between group sessions) and review the actions and results	4 sessions
Group termination or continuation and future plan	• Preparation for group termination or continuation • Future plan for maintaining mental well-being and positive caregiving experience	• Preparation for and discussion on termination issues e.g. separation anxiety, independence, and continuous use of coping and stress management skills learned • Review on learning experiences, challenges and goals/tasks achievement • Discussion on the continuity of caregiving, self-efficacy and development after completing the program; and appropriate help-seeking and use of community resources • Explanation of post-intervention assessments and follow-up taken in the coming 48 months • Exploration of continuation of group meetings and supports among members	2 sessions

a *The family-led mutual support group (FMSG) was modified from our previous family intervention program for people with schizophrenia (24, 25, 34)*.

b *The FMSG program was held bi-weekly with 16 sessions for 9 months*.

The family participants were highly encouraged to select the topics of their interest and modified the protocol to meet their needs. With group consensus, they identified definite tasks and focused/in-depth discussion for each group session. To facilitate the development of FMSG, the peer leaders worked with other group members to develop the group based on six principles of a successful family group program. These six principles included: disclosure of information with trust and respect; fostering dialectical and critical appraisal process (e.g., assisting and encouraging to think/consider alternatives for problem-solving); facilitating and protecting discussions about their taboo areas; fostering ‘All-in-Same-Boat’ feeling and “Working against a common plight”; encouraging mutual sharing and support; and offering opportunities to focus and resolve unique individual problems (35, 36).

Similarly, the family caregivers in the psychoeducation group program participated in 16 biweekly 2 h group sessions (14–16 members per education group), in addition to routine outpatient care. The program adopted the psychoeducation group manual established by McFarlane et al. (37) and Lehman et al.'s PORT programme (38). Participants in this group program received education and psychological support by one psychiatric nurse specialist (for 5 years) who was experienced in mental health education, rehabilitation and group therapy and trained by the research team with a 3-day (20 h) workshop. The training workshop consisted of mini-lectures, video watching and discussion, experience sharing and supervised practices of group leadership and facilitation. A few important topics were emphasized in this program: harmonious family relationships and environment, caregiving roles and demands, understanding about psychosis and its treatments and services, effective coping and communication skills, and problem-solving and crisis intervention skills in caregiving. The program consisted of five themes: introduction, engaging, and goal orientation (two sessions); mental health promotion, survival, and stress management skills training (five sessions); establishing a therapeutic family environment (two sessions); relapse prevention and resilience enhancement via problem-solving, interpersonal and life skills training (five sessions); and review and evaluation of learning on knowledge and skills and setting up future plans (two sessions).

Participants who completed >7 (out of 16) sessions were considered to be completers of the FMSG or psychoeducation group interventions and hence met the minimum per-protocol attendance threshold. All patients in both the FMSG and psychoeducation group programs were invited and encouraged to attend three sessions (between sessions 3 and 8) in which knowledge of the illness and treatment, medication adherence, and community mental healthcare services were introduced and discussed. An expert panel of eight members (two psychiatrists, nurse specialists, clinical psychologists, and ex-patients with early psychosis) validated the manuals of the FMSG and psychoeducation programs and independently assessed the clarity, relevance and appropriateness of their topics/contents. The majority of the items or topics in the two program manuals were rated as very clear/highly relevant (90–95%) and appropriate (93–98%) to the intervention. Only three items in each program were amended on the wordings and expressions to clarify their meanings and relevance. In addition, intervention progress, and fidelity monitoring was made by reviews of the audio-tapes of all group sessions of the two interventions. Program fidelity was assessed by two researchers independently with a checklist from the National Institute of Health Behavior Change Consortium (39) on the adherence to main topics or items, and instructions provided.

Participants in the TAU only (and also in the FMSG and psychoeducation group) received their routine psychiatric outpatient care. The routine care received was similar in the three clinics, consisting of psychiatric consultations, treatments and service referrals by psychiatrists, nursing education and advice on accessible mental healthcare services, home visits, and family assessments by healthcare workers, and social welfare, and family/individual counseling by medical social workers.

### Outcome Measures

For baseline measurement and post-tests, the family caregivers in this study were asked to complete the Family Assessment Device (FAD) for family functioning, Family Burden Interview Schedule (FBIS) for perceived caregiving burden and Family Support Services Index (FSSI) for utilization of services. They also completed a demographic data sheet at baseline. Their patients completed the Specific Level of Functioning Scale (SLOF) for patient functioning. Patients' psychotic symptoms were assessed with the Positive and Negative Syndrome Scale (PANSS) by their attending psychiatrist who was blinded to their group assignment. These Chinese and English versions of outcome measures were tested in Chinese populations with schizophrenia or psychotic disorders, indicating very satisfactory reliabilities and validities (24–26, 29, 40). Although the above outcome measures were self-rated by the participants themselves, the inter-rater reliabilities of these instruments were established between the outcome assessors (research assistant and first author), with intra-class correlations between 0.71 and 0.80 (*p* = 0.01–0.008).

Patients' number (frequency) and duration of psychiatric re-hospitalizations, and the total patients (per group) hospitalized and dosages and types of anti-psychotic drugs used in the past 6 months, were examined from the electronic patient records in the clinics. Dosages of antipsychotics were converted into haloperidol equivalents for comparison purposes (41).

#### Family Functioning

The 60-item FAD assessed multiple dimensions of family functioning among patients with severe mental illness (42). Items were rated on a four-point Likert scale (from 1-“strongly disagree” to 4-“strongly agree”).

#### Family Caregiving Burden

The 25-item FBIS is a semi-structured interview schedule used to assess the family's burden of care in schizophrenia (43, 44). It consists of six domains, including family finance, routine, leisure, interaction, physical health, and mental health, rated on a three-point Likert-type scale (0-“No burden,” 1-“Moderate burden,” and 2-“Severe burden”). A higher score indicated more severe caregiving burden.

#### Use of Family Services

The 16-item FSSI is a checklist (Yes/No response) to measure the utilization of community mental healthcare services commonly available/accessible to families of people with severe mental illness (45).

#### Patient Functioning

The 43-item SLOF was used to assess the level of psychosocial functioning of people with psychotic disorders, consisting of three functional areas (self-maintenance, social functioning and community living skills) (46). Items were rated on a five-point Likert scale (from 1-“totally dependent” to 5-“highly self-sufficient”), with a higher score indicating a better functioning.

#### Psychotic Symptoms

The 30-item PANSS is a universal measure assessing the symptom severity in psychotic disorders (47). Items were rated on an eight-point Likert scale, 0-“absent” to 7-“extreme,” with a higher score indicating more severe psychotic symptoms.

### Statistical Analyses

Based on intention-to-treat principle, all statistical analyses were conducted using the SPSS (IBM) for Windows, version 22.0. Analysis of variance or Kruskal-Wallis test by ranks was used to test any differences on demographic characteristics and outcome mean scores of the three study groups at baseline. Any of these variables found significant different between groups would be set as co-variant(s) in the outcome analyses. The baseline mean scores of all outcome variables were moderately correlated (Pearson's correlations between 0.38 and 0.60). With very minimal violation of the principles of multivariate analyses such as multivariate normality, equality of variance-covariance and outliers, mixed-model multivariate analysis of variance (MANOVA) test was performed to examine the interaction (Group × Time) treatment effects within- and between-group on all outcome variables across six measurements (48). This controlled trial recruited an adequate sample size for multivariate analyses on the six outcome variables. Only three univariate outliers were found in the outcome variables and these did not have extreme (very high/low) values. There was only one value (22.87) from all six outcomes with a Mahalanobis distance higher than the critical value of 22.46, thus indicating all outcome data had satisfactory multivariate normality. The scatterplots of the six outcomes for two study groups did not show any evidence of non-linearity. There were also moderate correlations between all outcome variables at baseline and first and second post-tests (Pearson's correlations between 0.38 and 0.60), showing very low chance of multi-collinearity among the outcomes. Box's M Test of Equality of Covariance Matrices and Levene's Test of Equality of Error Variances values were 6.48 (*p* = 0.231) and 0.12–0.23 (i.e., *p* < 0.05), indicating very satisfactory homogeneity of variance-covariance and equality of variance for the six outcomes. Only a few missing data (<5%) were found, and in accordance with intention-to-treat analyses these values were imputed using the initial data brought forward method (48), which had made little difference to the results.

With significant MANOVA results, the repeated-measures ANOVA test results were examined to identify between- and within-group differences on individual outcomes (FAD, FBIS, FSSI, SLOF, re-hospitalization rates, and PANSS), as well as the dosages of antipsychotic medication across measurements. For those outcomes with significant between-group differences, Helmert's contrasts test was then used to examine any significant differences in the means of individual outcome measures between groups at all post-tests. Differences on study outcomes in the FMSG indicating significant treatment effects were examined between the three clinics, between low attendees or in-completers (<8 sessions), borderline completers (8–10 sessions) and high attendees (≥11 sessions) in the FMSG, and between groups of total number of patients ever re-hospitalized in the past 6 months. These between group comparisons across all measurement points were conducted with ANOVA (followed by *t*-test) or Kruskal-Wallis test (followed by Mann-Whitney *U*-test). The level of statistical significance was set at 5%.

## Results

### Sample Characteristics and Baseline Outcome Scores

The socio-demographic characteristics of the study participants are summarized in Table 2. In the three study groups, mean ages of the caregivers were from 39.6 (*SD* = 8.9) to 42.1 (*SD* = 8.6) years (range 24–61 years) and two-thirds (63–67%) were female. Their relationships with patients were mainly parent (31–36%), child (23–26%), and spouse (29–33%). Mean ages of the patients were from 26.2 (*SD* = 7.3) to 28.9 (*SD* = 6.9) years (range 21–44 years) and more than half (51–54%) of them were male. Majority of the patients (53–60%) were taking low to medium dosages of oral anti-psychotics (Mean haloperidol equivalent values = 7.3–9.8 mg/day, *SD* = 3.9–5.0); mean duration of illness was 21–23 months (*SD* = 9.4–10.1, range 6–60). There were no significant differences in any of the socio-demographic characteristics of both caregivers and patients between the three groups (*p* > 0.11). There were also no significant differences in types/dosages of antipsychotics taken by the patients between groups (*p* = 0.20 and 0.33).

**Table 2:** Socio-demographic characteristics of family participants at baseline (*N* = 210).

**Characteristics**	**FMSG (*n* = 70)^**a**^f (%)**	**Psychoeducation (*n* = 70)^**a**^ f (%)**	**TAU (*n* = 70)^**a**^f (%)**	**ANOVA/KW test value^**b**^**	***p***
**FAMILY CAREGIVERS**					
Gender					
Male	26 (37.1)	24 (34.3)	23 (32.9)	1.80	0.20
Female	44 (62.9)	46 (65.7)	47 (67.1)		
Age	M = 41.1, *SD* = 6.3	M = 42.1, *SD* = 8.6	M = 39.6, *SD* = 8.9	2.13	0.16
20–29	20 (28.6)	19 (27.1)	21 (30.0)		
30–39	29 (41.4)	30 (42.9)	30 (42.9)		
40–49	14 (20.0)	15 (21.4)	14 (20.0)		
50 or above	7 (10.0)	6 (8.6)	5 (7.1)		
Education level					
Primary school or below	10 (14.3)	11 (15.7)	12 (17.1)	1.34	0.25
Secondary school	45 (64.3)	43 (61.4)	45 (64.3)		
University or above	15 (21.4)	16 (22.9)	13 (18.6)		
Relationship with patient					
Child	16 (22.9)	18 (25.7)	16 (22.9)	1.88	0.19
Parent	25 (35.7)	23 (32.9)	22 (31.4)		
Spouse	22 (31.4)	20 (28.6)	23 (32.9)		
Others (e.g., sibling and grandparent)	7 (10.0)	9 (12.8)	9 (12.8)		
Monthly household income (HK$)^c^	M = 18,730, *SD* = 4,585	M = 17,500, *SD* = 4,980	M = 18,330, *SD* = 4,950	1.78	0.17
5,000–10,000	10 (14.2)	9 (12.9)	10 (14.2)		
10,001–15,000	24 (34.3)	23 (32.8)	20 (28.6)		
15,001–25,000	27 (38.6)	29 (41.4)	31 (44.3)		
25,001–35,000	9 (12.9)	9 (12.9)	9 (12.9)		
**PATIENTS**					
Gender					
Male	38 (54.3)	37 (52.9)	36 (51.4)	1.40	0.23
Female	32 (45.7)	33 (47.1)	34 (48.6)		
Age	M = 26.8, *SD* = 6.5	M = 26.2, *SD* = 7.3	M = 28.9, *SD* = 6.9	1.56	0.22
21–29	40 (57.1)	42 (60.0)	38 (54.3)		
30–39	21 (30.0)	19 (27.1)	25 (35.7)		
40–49	9 (12.9)	9 (12.9)	7 (10.0)		
Duration of illness (months)	M = 20.5, *SD* = 9.8	M = 21.8, *SD* = 10.1	M = 23.0, *SD* = 9.4	1.78	0.19
6-12	20 (28.6)	19 (27.1)	18 (25.7)		
13-24	23 (32.9)	22 (31.4)	23 (32.9)		
25-36	14 (20.0)	16 (22.9)	15 (21.4)		
37-48	8 (11.4)	7 (10.0)	9 (12.9)		
48-60	5 (7.1)	6 (8.6)	5 (7.1)		
Mental condition^d^				2.50	0.11
Worsened	15 (21.4)	13 (18.6)	17 (24.3)		
Stable	40 (57.1)	39 (55.7)	38 (54.3)		
Improved	15 (21.4)	18 (25.7)	15 (21.4)		
Types of oral anti-psychotics				1.67	0.20
First generation	20 (28.5)	18 (25.7)	17 (24.3)		
Second generation	30 (42.9)	31 (44.3)	31 (42.9)		
Others (e.g., Reserpine)	10 (14.3)	9 (12.9)	9 (12.9)		
Combined modes	10 (14.3)	12 (17.1)	13 (18.6)		
Dosage of medication^e^	M = 7.34, *SD* = 3.87	M = 8.38, *SD* = 4.58	M = 9.76, *SD* = 4.95	1.12	0.33
High	10 (14.3)	9 (12.9)	11 (15.7)		
Medium	47 (67.1)	48 (68.5)	45 (64.3)		
Low	13 (18.6)	13 (18.6)	14 (20.0)		

FMSG, Family-led Mutual Support Group Program; TAU, Treatment-As-Usual only group

a *Frequency and percentage, f (%) or Mean and standard deviation, M and SD*.

b *ANOVA, F_(2, 268)_ or KW, Kruskal-Wallis test by ranks (H statistic, df = 2) was used to compare the socio-demographic variables between three groups*.

c US$1 = HK$7.8

d *Family caregiver's rating of patient's mental condition in the previous month, compared with that in the past year*.

e *Dosage levels of anti-psychotic medications for psychotic patients in haloperidol equivalent mean values (65)*.

Seven families declined to complete outcome measures or were lost to follow-up (mainly at 12-month or Time 3 and 48-month or Time 5 post-intervention) and five families withdrew from the study (i.e., three during the interventions and two at Time 5). Sixty-one participants (87.1%) in the FMSG and 59 (84.3%) in the family psycho-education group completed the intervention (i.e., attended >7 sessions, including those borderline completers and high attendees). Based on intention-to-treat principle, 205 participants were included in the outcome analyses. The main reasons for drop-out and/or low attendance rate included: time constraints due to demands for caregiving and household chores (*n* = 4); unstable or worsened mental state of patients (*n* = 4); not interested or supported (*n* = 4); and felt the interventions were not helpful in meeting caregiving needs (*n* = 4). Mean and median group attendance in the FMSG was 10.2 (*SD* = 4.3, media *n* = 9, range 5–16 sessions) and in the psychoeducation group was 9.1 (*SD* = 4.8, media *n* = 8, range 4–16 sessions).

Intervention fidelities for the FMSG were between 78.3 and 84.1% (81.2% in average) and for the psychoeducation group were between 91.5 and 96.2% (94.5% in average). These fidelity scores revealed a very satisfactory level of adherence to the structured psychoeducation group protocol, and only fairly satisfactory adherence to the member-driven FMSG protocol.

Baseline mean scores of the outcome variables are summarized in Table 3. All of them indicated no significant differences (*p* > 0.2) between groups, indicating homogeneity of study groups at pre-test.

**Table 3:** Mean outcome scores at all five measurements and their individual interactive (group x time) treatment effects (*N* = 205).

**Measures**	**FMSG (*****n*** **= 69)**		**Psychoeducation (*****n*** **= 68)**		**TAU (*****n*** **= 68)**		**F_**(2, 203)**_, P**	**Effect size partial [eta]^**2**^**
	**M**	**SD**	**M**	**SD**	**M**	**SD**		
FAD (0-50)^a^							8.13, 0.003	0.41
Time 1	23.82	6.13	22.93	7.32	24.88	8.72		
Time 2	26.13	7.01	26.01	8.81	21.21	8.91		
Time 3	29.03	9.93	25.12	9.22	22.52	9.12		
Time 4	28.02	8.19	24.08	10.77	21.97	9.98		
Time 5	30.12	11.20	26.02	12.89	23.12	10.23		
FBIS (0-50)							7.21, 0.004	0.35
Time 1	30.01	7.12	30.98	6.45	31.65	7.77		
Time 2	27.13	8.01	28.12	8.92	30.08	8.91		
Time 3	25.05	9.13	26.05	8.73	30.12	9.65		
Time 4	22.12	10.01	25.63	9.98	29.22	9.13		
Time 5	19.11	10.98	27.01	8.92	30.98	11.21		
SLOF (43-215)							6.51, 0.005	0.29
Time 1	139.33	19.32	140.11	12.89	139.98 20.01			
Time 2	156.12	22.10	145.93	18.92	135.01 22.45			
Time 3	168.92	25.01	148.91	20.12	132.45 20.01			
Time 4	178.12	29.13	154.89	23.12	136.92 27.81			
Time 5	192.10	28.02	164.12	20.33	140.33 22.67			
**PANSS (30-210)**								
Total score							5.02, 0.005	0.28
Time 1	97.12	10.01	97.67	9.98	97.12	10.38		
Time 2	82.34	14.23	86.29	10.33	98.86	14.59		
Time 3	74.11	15.67	76.56	11.45	92.12	16.12		
Time 4	60.23	14.12	77.11	12.78	101.10	20.66		
Time 5	60.87	16.34	75.55	14.38	97.65	19.87		
Positive symptoms							5.13, 0.005	0.30
Time 1	28.11	8.64	28.68	9.12	28.00	9.87		
Time 2	23.01	8.92	23.65	8.13	30.22	10.01		
Time 3	19.13	8.32	19.81	7.23	26.89	11.10		
Time 4	16.18	7.65	20.33	9.77	33.91	13.22		
Time 5	15.86	8.91	18.58	8.38	29.50	10.57		
Negative symptoms							2.42, 0.09	0.08
Time 1	21.98	9.01	22.02	8.56	21.01	8.12		
Time 2	19.79	8.20	20.13	9.95	23.91	9.92		
Time 3	17.92	9.33	18.34	8.73	20.33	8.72		
Time 4	18.01	7.02	20.01	9.77	24.01	7.71		
Time 5	17.82	8.34	17.11	8.22	20.82	9.69		
FSSI (1-16)							2.34, 0.12	0.05
Time 1	4.32	1.15	4.58 1.02	5.01	1.33			
Time 2	4.91	1.33	4.95 1.42	5.12	1.02			
Time 3	4.60	1.44	5.01 1.88	4.52	1.78			
Time 4	3.87	1.81	4.39 1.75	4.21	1.88			
Time 5	4.00	1.22	4.42 1.98	4.40	1.73			
**RE-HOSPITALIZATIONS (LAST 6 MONTHS)**								
Frequency/number							6.51, 0.005	0.31
Time 1	2.52	1.01	2.62	1.01	2.81	1.12		
Time 2	2.01	0.98	2.59	1.00	2.91	1.33		
Time 3	1.81	0.88	2.33	1.22	3.22	1.24		
Time 4	1.75	0.91	2.67	1.02	3.30	1.49		
Time 5	1.33	1.03	2.88	1.17	3.00	1.50		
**LENGTH / DURATION (DAYS)**^**b**^								
Time 1	19.88	7.98	20.11	6.11	19.22	7.33	2.68. 0.10	0.08
Time 2	16.33	9.13	17.22	8.45	22.11	9.34		
Time 3	19.12	10.22	20.34	6.23	19.12	10.11		
Time 4	17.12	10.01	20.12	9.33	19.91	9.88		
Time 5	21.23	11.56	22.11	13.75	24.11	11.32		

*FMSG, Family-led Mutual Support Group Program; TAU, Treatment-As-Usual only*.

*FAD, Family Assessment Device; FBIS, Family Burden Interview Schedule; FSSI, Family Support Service Index; SLOF, Specific Level of Functioning; PANSS, Positive and Negative Symptoms Scale*.

*Time 1, baseline measurement; Time 2, 1 week post-intervention; Time 3, 12 months post-intervention; Time 4, 24 months post-intervention; Time 5, 48 months post-intervention*.

a *Possible range of scores of each of the outcome instruments in parenthesis*.

b *Average duration or length of re-admissions into a psychiatric in-patient hospital/unit, in terms of average days of hospital-stay per month in the past 4-6 months at five measurements (i.e., baseline and 4 post-tests)*.

### Treatment Effects

In Table 3, the results of means and standard deviations of the outcome measures at baseline (Time 1) and four post-tests (Times 2–5) are summarized. The results of mixed-model MANOVA test indicated there was a statistically significant interactive (Group × Time) effect on the combined set of seven outcome variables, *F*_(7, 205)_ = 29.1, *p* = 0.001 (Wilks' Lambda = 0.81; a large effect with partial [eta]^2^ = 0.38). The results of between-group ANOVA tests for individual outcomes across five measurements (Table 3), indicated that there were statistically significant differences between the three groups on the caregivers' FAD score [*F*_(2, 204)_ = 8.13, *p* = 0.003] and FBIS score [*F*_(2, 204)_ = 7.21, *p* = 0.004 and patients' SLOF score [*F*_(2, 203)_ = 6.51, *p* = 0.005], PANSS score [*F*_(2, 203)_ = 5.02, *p* = 0.005, and number of re-hospitalizations [*F*_(2, 203)_ = 6.51, *p* = 0.005]. Their effect sizes in terms of partial [eta]^2^ ranged 0.28–0.41 (see Table 3).

Helmert's contrasts tests results (Table 4) showed that the FMSG participants had significantly greater improvements in the below outcomes than the other two study groups at different post-tests:

- Family functioning (FAD score) at Times 3–5 than the TAU; and at Times 4–5 than the family psychoeducation group; whereas, the psychoeducation group also indicated greater improvement in family functioning than the TAU at Time 2.
- Family burden (FBIS score) at Times 2–5 than the TAU; and at Times 4–5 than the psychoeducation group; whereas, the psychoeducation group also indicated greater reduction in burden than the TAU at Times 3–5.
- Patient functioning (SLOF score) at all post-test (Times 2–5) than both the TAU and psychoeducation group. The SLOF scores increased progressively in the psychoeducation group over the follow-ups, indicating significant greater improvement than the TAU at all post-tests.
- Psychotic symptoms in overall score and positive symptoms at all post-tests (Times 2–5) than the TAU and only in overall score at Times 4–5 than the psychoeducation group. Whereas, the psychoeducation group also indicated greater improvement in psychotic symptoms than the TAU at all post-tests.
- Average number of re-hospitalizations (reduced) at Times 2–5 than the TAU; and at Times 4–5 than the psychoeducation group.

**Table 4:** Helmert's contrasts test results of study outcomes with significant between-group differences.

**Measures**	**Time 2**			**Time 3**			**Time 4**			**Time 5**		
	**MD, SE**	**T, p**	**95% CI**	**MD, SE**	**T, p**	**95% CI**	**MD, SE**	**T, p**	**95% CI**	**MD, SE**	**T, p**	**95% CI**
**FAD**												
FMSG vs. TAU	3.92, 1.81	2.83, 0.08	3.25-4.61	6.51, 0.83	*4.52, 0.03*	5.83-8.21	6.05, 1.90	*4.23, 0.04*	5.23-6.78	7.00, 1.02	*5.01, 0.01*	6.23-7.71
FMSG vs. Psychoeducation	0.12, 0.48	0.55, 0.30	−0.28-0.79	3.91, 0.82	2.80, 0.09	3.10-4.78	3.94, 1.98	*3.12, 0.05*	2.98-5.02	4.10, 1.50	*3.34, 0.04*	2.78-5.42
Psychoeducation vs. TAU	4.80, 0.35	*3.78, 0.03*	4.18-5.53	2.60, 0.25	1.34, 0.12	2.15-2.91	2.11, 0.85	1.23, 0.16	1.65-3.78	2.90, 1.31	1.56, 0.10	1.58-4.21
**FBIS**												
FMSG vs. TAU	2.95, 0.88	3.10, 0.05	1.98-3.81	5.07, 0.78	5.33, 0.007	4.21-5.86	7.10, 1.01	*8.13, 0.005*	5.91-8.21	11.87, 0.45	*10.12, 0.001*	10.56-2.23
FMSG vs. Psychoeducation	0.99, 0.80	0.89, 0.20	0.20-1.69	1.00, 0.50	0.90, 0.21	0.48-1.52	3.51, 0.35	*3.56, 0.04*	3.18-3.93	7.90, 1.02	*8.25, 0.005*	6.90-8.94
Psychoeducation vs. TAU	1.96, 0.05	1.68, 0.10	1.76-2.28	4.07, 0.88	4.13, 0.01	3.18-4.92	3.59, 0.82	*3.58, 0.04*	2.78-4.33	3.97, 3.02	*3.82, 0.03*	1.01-6.83
**SLOF**												
FMSG vs. TAU	21.11, 0.51	*9.21, 0.005*	20.23-21.98	36.47, 4.98	*14.21, 0.001*	31.45-41.26	41.20, 2.13	*16.98, 0.001*	38.91-43.40	51.77, 6.13	*18.95, 0.001*	48.45-57.93
FMSG vs. Psychoeducation	10.19, 4.12	*7.10, 0.01*	6.10-15.23	20.01, 4.88	*9.01, 0.005*	15.89-24.91	23.23, 6.01	*9.43, 0.004*	16.87-29.30	27.98, 7.95	9.81, 0.003	20.13-25.13
Psychoeducation vs. TAU	10.92, 4.01	*7.25, 0.01*	6.82-15.13	16.46, 0.10	*8.15, 0.007*	16.33-16.58	17.97, 4.56	*8.35, 0.006*	13.42-22.28	23.79, 2.50	*9.35, 0.004*	21.02-26.30
**PANSS**												
FMSG vs. TAU	16.52, 0.41	*4.79, 0.01*	16.00-17.01	18.01, 1.01	*4.98, 0.01*	16.91-19.34	40.87, 6.34	*9.13, 0.001*	33.68-47.12	36.78, 3.12	*8.71, 0.003*	32.45-39.91
FMSG vs. Psychoeducation	3.95, 3.01	1.89, 0.10	0.91-5.98	2.45, 3.01	1.12, 0.13	−0.91-5.32	16.88, 1.98	*4.70, 0.01*	14.46-18.51	14.68, 1.96	*4.53, 0.02*	12.71-16.60
Psychoeducation vs. TAU	12.57, 4.12	*4.33, 0.03*	8.40-16.71	15.52, 4.89	*4.26, 0.03*	11.54-20.11	23.99, 7.12	*5.34, 0.007*	16.48-31.08	22.11, 5.45	*5.22, 0.008*	16.86-27.56
**NUMBER OF RE-HOSPITALIZATIONS**^**a**^												
FMSG vs. TAU	0.90, 0.42	*4.12, 0.04*	0.48-1.34	1.41, 0.42	*6.13, 0.01*	1.01-1.86	1.55, 0.50	*6.25, 0.01*	1.04-2.06	1.67, 0.45	*6.38, 0.009*	1.20-2.08
FMSG vs. Psychoeducation	0.58, 0.03	1.12, 0.15	0.54-0.64	0.52, 0.40	1.20, 1.10	0.16-0.94	0.92, 0.09	*4.10, 0.05*	0.86-1.04	1.55, 0.15	*6.19, 0.01*	1.30-1.70
Psychoeducation vs. TAU	0.32, 0.30	0.98, 0.20	0.03-0.54	0.89, 0.02	4.03, 0.06	0.85-0.94	0.73, 0.30	3.75, 0.08	0.32-1.08	0.12, 0.32	0.82, 0.28	−0.24-0.46

*FMSG, Family-led Mutual Support Group Program; TAU, Treatment-As-Usual only*.

*FAD, Family Assessment Device; FBIS, Family Burden Interview Schedule; SLOF, Specific Level of Functioning; PANSS, Positive and Negative Symptoms Scale*.

*Time 2, 1 week post-intervention; Time 3, 12 months post-intervention; Time 4, 24 months post-intervention; Time 5, 48 months post-intervention*.

*MD, Mean score difference of an outcome measure between groups; SE, Standard error of mean difference; 95%CI, 95% Confidence Interval of mean difference*.

*T-test (2-tailed) was to compare the mean differences between two groups at each of the four post-tests and significant results are in an italic mode*.

a *Average number of re-admissions into a psychiatric in-patient hospital/unit in the past 4-6 months at five measurements*.

In addition, the FMSG showed significantly less patients re-admitted into a psychiatric unit over the past 6 months than those in the psychoeducation group and TAU at Times 3–5 [Kruskal Wallis test value = 12.33, *p* = 0.005; at Time 3, 19 (27%) vs. 25 (37%) in psychoeducation group vs. 28 (41%) in TAU; at Time 4, 11 (16%) vs. 18 (26%) in psychoeducation group vs. 22 (32%) in TAU; and 10 (14%) vs. 18 (26%) vs. 25 (37%), accordingly]. However, service utilization (FSSI score) ranged from 3.87 (*SD* = 1.81) to 5.12 (*SD* = 1.02) in the three groups over the 48-month follow-up, indicating no significant changes in demands for these family supporting services over time (*p* > 0.12). Otherwise, there were no significant differences on the above significant outcomes between three clinics (*p* = 0.10–0.19) and between in-completers (<8 sessions), borderline completers (8–10 sessions) and high (≥11 sessions) attendees (*p* = 0.09–0.25, using Kruskal-Wallis test; see detailed results in Table 5) in the FMSG, and on patients' types and dosages of anti-psychotics between groups over time (*p* > 0.10).

**Table 5:** Mean and median outcome scores for completers and in-completers of the FMSG and their comparisons using Kruskal-Wallis test (*N* = 69).

**Measures**	**In-completers (*****n*** **= 8)**			**Borderline completers (*****n*** **= 21)**			**High attendees (*****n*** **= 40)**			**Kruskal-Wallis test value, *P***
	**M**	**SD**	**Median (range)**	**M**	**SD**	**Median (range)**	**M**	**SD**	**Median (range)**	
FAD (0-50)^a^										6.13, 0.11
Time 1	23.03	5.02	22.62 (16.85–25.71)	23.93	6.39	24.53 (19.82–28.04)	23.88	5.85	24.94 (20.85–27.93)	
Time 2	25.71	7.20	24.23 (17.12–28.95)	26.01	8.33	23.92 (17.93–28.27)	26.98	8.70	27.66 (21.92–31.63)	
Time 3	27.56	8.02	25.43 (23.43–30.12)	28.78	9.45	27.23 (23.84–31.13)	29.32	9.71	28.53 (22.16–32.84)	
Time 4	27.41	10.21	26.84 (22.11–32.13)	27.23	10.01	28.84 (21.82–31.93)	28.89	9.05	28.05 (23.85–34.53)	
Time 5	27.87	11.20	26.65 (20.21–31.67)	29.45	10.78	28.64 (23.57–34.63)	30.48	10.51	29.94 (21.85–37.46)	
FBIS (0–50)										7.11, 0.09
Time 1	29.80	6.78	30.64 (23.82–34.94)	29.91	6.81	28.26 (20.14–31.93)	30.33	6.02	29.62 (20.01–29.84)	
Time 2	27.96	8.43	28.95 (20.81–33.55)	27.46	8.03	26.85 (20.80–32.54)	27.03	8.76	27.03 (17.27–26.82)	
Time 3	25.98	9.71	26.65 (19.25–30.84)	25.11	8.98	24.64 (20.10–29.35)	24.81	8.60	23.54 (19.81–27.03)	
Time 4	24.34	10.21	25.44 (18.88–31.12)	23.68	9.12	25.06 (19.60–30.73)	21.90	9.98	22.26 (16.64–24.92)	
Time 5	21.33	10.45	22.65 (16.15–27.98)	19.58	9.98	20.23 (17.30–27.26)	19.02	11.20	20.17 (15.13–24.86)	
SLOF (43–215)										4.89, 0.13
Time 1	140.31	14.54	138.61 (116.85–145.72)	138.12	15.34	139.64 (126.53–149.82)	140.35	17.29	139.29 (117.22–156.75)	
Time 2	154.11	20.01	151.13 (136.15–178.14)	157.90	19.32	160.33 (120.80–179.32)	158.33	20.34	160.11 (129.81–185.92)	
Time 3	162.88	24.78	157.22 (120.82–171.23)	167.33	22.12	170.12 (144.20–189.24)	170.55	22.71	138.65 (116.81–145.72)	
Time 4	165.10	28.11	158.03 (111.24–175.91)	179.33	25.33	175.04 (118.81–198.13)	180.22	20.88	182.49 (135.05–199.45)	
Time 5	170.19	29.87	168.54 (114.91–179.62)	190.10	23.30	188.01 (129.50–199.81)	193.13	25.77	194.82 (140.89–221.81)	
PANSS (30–210)										
Total score										5.98, 0.12
Time 1	99.38	9.33	98.61 (81.85–107.53)	98.67	9.02	97.55 (75.80–108.34)	97.12	11.45	98.22 (77.71–108.86)	
Time 2	86.58	11.22	87.63 (81.82–107.51)	82.77	12.44	83.91 (61.77–97.50)	82.10	14.59	81.46 (69.65–96.12)	
Time 3	77.82	17.55	75.32 (65.11–88.92)	75.11	13.56	77.44 (62.89–96.22)	72.80	15.67	73.23 (61.99–79.88)	
Time 4	68.20	16.44	65.11 (59.88–79.45)	61.12	10.56	60.15 (53.01–84.65)	60.05	12.71	62.65 (50.33–79.25)	
Time 5	67.55	15.22	69.13 (51.90–87.23)	62.02	13.65	64.87 (51.67–87.22)	59.66	13.81	57.86 (50.31–68.83)	
Positive symptoms										5.01, 0.14
Time 1	27.93	7.60	27.36 (24.31–29.98)	29.33	8.23	29.21 (21.31–27.98)	28.56	8.71	28.00 (23.25–31.57)	
Time 2	23.54	7.88	23.39 (19.26–28.75)	23.55	9.13	24.55 (19.48–28.67)	22.99	9.31	23.48 (19.45–26.79)	
Time 3	21.43	10.32	24.12 (19.22–30.44)	20.21	7.88	21.87 (18.12–26.54)	19.00	9.13	18.92 (15.11–26.15)	
Time 4	19.27	10.65	20.89 (16.58–28.83)	18.13	8.44	19.23 (16.33–25.12)	16.01	8.72	17.58 (13.22–23.78)	
Time 5	18.71	10.90	19.94 (14.71–27.69)	17.08	7.23	18.81 (16.51–24.92)	15.19	7.22	15.96 (13.01–20.68)	
Negative symptoms										4.42, 0.17
Time 1	21.43	7.88	22.31 (19.44–27.12)	22.00	7.12	23.00 (20.12–26.43)	22.11	8.00	21.55 (18.76–25.41)	
Time 2	20.45	9.56	21.45 (16.45–28.76)	19.32	9.38	20.13 (18.24–25.89)	19.98	9.35	19.01 (15.31–26.78)	
Time 3	19.90	9.77	20.98 (16.21–28.13)	17.01	9.71	18.13 (16.30–25.13)	17.30	8.52	16.78 (12.88–20.66)	
Time 4	19.45	10.22	21.56 (16.00–29.02)	17.33	9.88	17.12 (14.20–24.02)	17.95	8.79	18.32 (13.22–25.02)	
Time 5	19.80	9.64	20.96 (17.21–28.31)	17.69	8.98	19.01 (15.03–25.47)	17.86	9.69	18.61 (13.54–24.25)	
FSSI (1–16)										3.02, 0.25
Time 1	4.23	1.01	4.08 (3.23–5.45)	4.30	1.08	4.41 (3.01–5.11)	4.58	1.02	4.48 (3.12–5.61)	
Time 2	4.90	1.10	4.50 (2.87–6.85)	4.96	1.12	4.65 (2.97–5.98)	4.85	0.98	4.79 (2.01–6.99)	
Time 3	4.23	1.27	4.51 (2.23–7.67)	4.76	1.18	4.99 (2.86–6.90)	4.60	1.12	4.59 (2.35–7.02)	
Time 4	3.80	1.22	4.22 (3.01–7.58)	3.98	1.31	3.88 (2.14–7.05)	3.88	1.33	3.78 (2.61–5.98)	
Time 5	4.10	1.33	4.30 (2.56–6.73)	3.89	1.11	4.15 (2.90–6.98)	4.00	1.15	4.10 (2.31–6.65)	
**RE-HOSPITALIZATIONS (LAST 6 MONTHS)**										
Frequency/number										5.27, 0.15
Time 1	2.60	1.11	2.58 (1.00–5.00)	2.68	1.04	2.89 (1.00–5.00)	2.65	1.01	2.80 (0.00–5.00)	
Time 2	2.10	0.99	2.01 (0.00–4.00)	2.08	1.22	2.21 (1.00–4.00)	2.01	1.22	2.00 (0.00–6.00)	
Time 3	1.99	1.42	2.25 (1.00–4.00)	1.81	1.32	1.98 (1.00–4.00)	1.80	1.33	2.00 (0.00–6.00)	
Time 4	1.90	1.33	2.35 (1.00–4.00)	1.89	1.21	1.78 (0.00–5.00)	1.72	1.19	1.82 (0.00–4.00)	
Time 5	1.93	1.35	2.15 (1.00–4.00)	1.88	1.33	2.98 (1.00–6.00)	1.30	1.11	1.40 (0.00–3.00)	

*FMSG, Family-led Mutual Support Group Program*.

*FAD, Family Assessment Device; FBIS, Family Burden Interview Schedule; FSSI, Family Support Service Index; SLOF, Specific Level of Functioning; PANSS, Positive and Negative Symptoms Scale*.

*Time 1, baseline measurement; Time 2, one week post-intervention; Time 3, 12 months post-intervention; Time 4, 24 months post-intervention; Time 5, 48 months post-intervention*.

a *Possible range of scores of each of the outcome instruments in parenthesis*.

## Discussion

The findings of this randomized controlled trial support that the 9-month family-led mutual support group program (FMSG) for people with recent-onset psychosis can significantly improve patients' mental state, re-hospitalization rates and functioning, in addition to improving family functioning and caregivers' perceived burden over a long-term (48 months) follow-up. This trial is one of very few to test the effects of a peer-led family support group in providing family-centered care for patients with early-stage psychosis in comparison to usual psychiatric outpatient care or a conventional family psychoeducation group program. The FMSG participants also did not show increased demands for community mental health services and uses (dosages) of anti-psychotic medications over the 48-month follow-up. Therefore, in terms of both primary and secondary outcomes, the findings highlight the potential benefits of applying this family-led mutual support group for Chinese people with recent-onset psychosis in community-based family-oriented mental health care.

The substantial positive effects of the peer-led FMSG over the 48-month follow-up provides strong evidence that this can be an effective approach to family intervention for people with early stage or recent-onset psychosis not only as evidenced in Western countries (19, 49), but also in Asian families with higher inter-dependence and collectivistic attitudes and behaviors in caregiving (25, 26). Its benefits are more significant and substantive than those of the commonly accepted family psychoeducation group intervention (14, 15). The results further support that the FMSG has sustainable effects for both psychotic patients and their families as suggested by previous controlled trials of professional-facilitated family mutual support group programs for schizophrenia with shorter periods of interventions and/or follow-ups (24, 25).

The results also provide evidence on the benefits of mutual support groups on family caregivers' burden of care and psychosocial health and well-being, as well as functioning of the whole family. These findings are less consistently found in previous family interventions for psychotic disorders (15, 19, 50). Recent research suggests that family-led mutual support groups can improve family functioning for two possible reasons. Firstly, enhanced knowledge, skills and social support for illness management results in better perceived controls over their family relationships and caregiving. Secondly, the “All-in-Same-Boat” feeling and shared belief among group members induces a strong commonality and sense of coherence, providing effective social learning and practical advice on effective caring strategies (19, 23). In addition, there were also significant and consistent improvements in the patients' psychosocial functioning, psychotic symptoms and number of re-hospitalizations, indicating that supporting family caregivers where an individual suffers from psychosis is essential and crucial to their recovery from the illness (51).

When compared with TAU, the conventional family psychoeducation group also demonstrated significant improvements in family burden and patient symptoms and functioning over the 48-month follow-up, as well as family functioning at one-week post-intervention. Nevertheless, the positive effects found in the psychoeducation group were not as large (effect sizes) and substantive as those in the FMSG. While psychoeducation group programs for families of people with schizophrenia and psychotic disorders are widely accepted and implemented in Western countries (8–10) and China (52), recent systematic reviews on family interventions (8–11) concluded that this approach can produce moderate but inconsistent benefits in families' burden, stress and illness management and well-being, especially in a longer term (>18 months) of follow-up. The findings of this study support a conclusion that family psychoeducation groups may be less likely to demonstrate sustainable benefits on family functioning at ≥12 months follow-up, as well as lower effects on different aspects of family and patient outcomes over 48 months post-intervention. Therefore, peer-led family mutual support groups can be considered a more effective approach to family intervention in recent-onset psychosis than the standardized psychoeducation group intervention.

In this study, the consistent and substantive symptom reduction (PANSS scores) and lower number of re-hospitalizations among the patients in the FMSG over the 4 years of follow-up may suggest much better illness/symptom management than those in both the TAU and psychoeducation group. While there have been increasing amounts of case controlled and prospective evaluation studies favoring the use of mutual support groups for patients with severe mental illness and their families, only few randomized controlled trials have been conducted to evaluate its effects or cost-benefits in community mental healthcare services (8–10, 15). In addition, the FMSG also demonstrated a stable or slightly reduced demand for community mental health services for patients and/or families and thus this approach might not result in any additional service needs or costs in the community.

The results of this controlled trial provide evidence on a less structured family-oriented, peer support or empowerment program for caregivers of people in early stage psychosis, which does not require a professional leader or therapist with extensive training and/or a highly structured therapy manual or intervention protocol. The FMSG can be led by the peer caregivers in a flexible, supportive and interactive mode. This “fluid” or dynamic approach may be more feasible in community-based interventions with resource constraints and result in support that is well focused or oriented to individual family needs (19, 21). Although this family-oriented mutual support group intervention may be more feasible and effective, the lack of a structured treatment manual may present challenges in faithfully replicating the intervention in future research. However, the FMSG clearly outlined the main topics and content for individual sessions for the participants to follow; and this might serve as a protocol or structure of the intervention for consistency of implementation and replication.

In this controlled trial, there were relatively low overall attrition rates (withdrawal or drop-out) of families (*n* = 12, 5.7%) and very high intervention completion rates (87% in FMSG and 84% in psychoeducation group). There were also very high completion rates of follow-up (*n* = 59–60 out of 70) in the three study groups. These findings could reflect that the participants perceived the mutual support in the FMSG as desirable and beneficial to their needs for caregiving and social support. Therefore, their study/intervention completions were higher than those (50–80%) in studies testing those commonly used approaches to family intervention in psychosis (8, 9, 12). However, a few other explanations could be given for such low attritions and/or non-completions in this trial. Firstly, the families who participated in the study could be highly motivated and enthusiastic toward patient care and recovery. They might actively engage in any interventions perceived as being potential helpful, as suggested in other studies of family support and interventions in early psychosis (25, 53, 54). Secondly, the patients were young, relatively mentally stable with low to moderate doses of anti-psychotic medications; and most families were supportive to their patients and were receiving an adequate monthly income. Lastly, the families might have been particularly motivated to attend because there are only few structured family intervention programs for recent-onset psychosis available in Hong Kong, or indeed worldwide (6, 10, 29, 55).

In traditional Chinese and Asian cultures, families are often reluctant to seek help from unfamiliar people due to concerns about exposing family affairs and difficulties to outsiders, or even some relatives (termed “*saving face*”) (26, 56). A higher reluctance to seek help can be found among Chinese families when faced with stigmatization toward mental illness (25, 57). If family intervention is to be effective in Chinese or Asian populations, it should be culturally sensitive. The substantive significant positive results of this study on improving family functioning and burden may suggest that the Chinese families in the FMSG benefited from the reciprocal sharing of information on effective caregiving, hence improving their passivity in seeking help throughout and after the groups. These findings can support the use of family-led mutual support group interventions in Chinese and Asian populations to facilitate their reciprocal learning and support in caring for a family member with severe mental illness, which they may not be able to obtain in a professional-led group intervention.

Recent practice guidelines (6, 10, 11) and systematic reviews (15, 21) have recommended that family intervention programs in psychosis should optimally have a long duration (e.g., >1 year), be facilitated by a trained professional and have content tailored for individual families' needs. However, the FMSG program in this trial provides a time-limited, flexible and interactive peer-led group environment over a 9-month duration, yet still has demonstrated very long-term significant positive effects on both patients' and families' psychosocial health outcomes, when compared to both the standard psychiatric care and standardized family psychoeducation group program.

However, the controlled trial has a few potential limitations. First, the study participants was recruited from three of 24 psychiatric clinics in the country and they were limited to those outpatients with 5 years of onset of psychosis. Therefore, these patients and their families might be relatively more optimistic and enthusiastic about patient recovery and independent living (1, 10), which could limit the generalizability of the findings. Second, the families were relatively well educated and financially comfortable, they also volunteered to participate and could be highly motivated, therefore they might not be representative to most families seeking or receiving community mental healthcare services. Third, although the FMSG program was a fairly structured intervention program, it was difficult to completely standardize because of the flexible member-agreed protocol and content. Similarly, the group dynamics and leadership skills of peer leaders (with less extensive training), as well as the interactions and instrumental support between group members outside those group sessions, were not standardized during the intervention. Hence, the consistency and integrity of the FMSG implementation could be the confounders for its treatment effects; and these intervention issues and their contributions to the positive effects of the FMSG should be investigated in future research.

In conclusion, the results of this trial have demonstrated that the family-led mutual support group (FMSG) for outpatients with recent-onset psychosis can be effective to improve both patient and family functioning and patient re-hospitalizations over a 4-year follow-up. There were also no increases in the demands for community mental health services among the families in the FMSG. The substantive and significant effects of this controlled trial may have strong implications for the design and practice of family intervention for people with recent-onset psychosis, focusing on social support, reciprocal learning and assistance, and empowerment as its main therapeutic elements. The positive study findings support the need for more research on this peer support group model in families of people with psychotic disorders with diverse socio-demographic and ethnic backgrounds, and their patients with wider illness characteristics and co-morbidities of other mental illnesses.

## Ethics Statement

The study protocol was approved by the Human Subjects Research Ethics Subcommittee of The Hong Kong Polytechnic University and The NTEC Cluster Research Ethics Committee of Hospital Authority, Hong Kong. Informed voluntary written consent was obtained from every individual participants who were screened for study eligibility. The study methods and reporting were performed according to the latest CONSORT 2010 guidelines.

## Author Contributions

WC and SC: study design; WC and SC: Funding acquisition; WC: Study coordination and implementation; WC, DB, and SC: Data collection and analysis; WC: Drafting the manuscript; All authors have reviewed and approved the final manuscript as submitted.

### Conflict of Interest Statement

The authors declare that the research was conducted in the absence of any commercial or financial relationships that could be construed as a potential conflict of interest.
